# Phylogeographic analysis reveals significant spatial genetic structure of *Incarvillea sinensis* as a product of mountain building

**DOI:** 10.1186/1471-2229-12-58

**Published:** 2012-04-30

**Authors:** Shaotian Chen, Yaowu Xing, Tao Su, Zhekun Zhou, Emeritus David L Dilcher, Douglas E Soltis

**Affiliations:** 1 Key Laboratory of Biodiversity and Biogeography, Kunming Institute of Botany, Chinese Academy of Sciences, Kunming, Yunnan 650204, China; 2 Key Laboratory of Tropical Forest Ecology, Xishuangbanna Tropical Botanical Garden, Chinese Academy of Sciences, Mengla, 666303, China; 3 Institute of Systematic Botany, University of Zürich, Zürich, 8008, Switzerland; 4 Department of Biology, Indiana University, Bloomington, IN 47405, USA; 5 Department of Biology, University of Florida, Gainesville, FL 32611-7800, USA

**Keywords:** Spatial genetic pattern, cpDNA variations, Phylogeography, Eastern Asian plant

## Abstract

**Background:**

*Incarvillea sinensis* is widely distributed from Southwest China to Northeast China and in the Russian Far East. The distribution of this species was thought to be influenced by the uplift of the Qinghai-Tibet Plateau and Quaternary glaciation. To reveal the imprints of geological events on the spatial genetic structure of *Incarvillea sinensis*, we examined two cpDNA segments ( *trn*H- *psb*A and *trn*S- *trnf*M) in 705 individuals from 47 localities.

**Results:**

A total of 16 haplotypes was identified, and significant genetic differentiation was revealed (*G*_ST_ =0.843, *N*_ST_ = 0.975, P < 0.05). The survey detected two highly divergent cpDNA lineages connected by a deep gap with allopatric distributions: the southern lineage with higher genetic diversity and differentiation in the eastern Qinghai-Tibet Plateau, and the northern lineage in the region outside the Qinghai-Tibet Plateau. The divergence between these two lineages was estimated at 4.4 MYA. A correlation between the genetic and the geographic distances indicates that genetic drift was more influential than gene flow in the northern clade with lower diversity and divergence. However, a scenario of regional equilibrium between gene flow and drift was shown for the southern clade. The feature of spatial distribution of the genetic diversity of the southern lineage possibly indicated that allopatric fragmentation was dominant in the collections from the eastern Qinghai-Tibet Plateau.

**Conclusions:**

The results revealed that the uplift of the Qinghai-Tibet Plateau likely resulted in the significant divergence between the lineage in the eastern Qinghai-Tibet Plateau and the other one outside this area. The diverse niches in the eastern Qinghai-Tibet Plateau created a wide spectrum of habitats to accumulate and accommodate new mutations. The features of genetic diversity of populations outside the eastern Qinghai-Tibet Plateau seemed to reveal the imprints of extinction during the Glacial and the interglacial and postglacial recolonization. Our study is a typical case of the significance of the uplift of the Qinghai-Tibet Plateau and the Quaternary Glacial in spatial genetic structure of eastern Asian plants, and sheds new light on the evolution of biodiversity in the Qinghai-Tibet Plateau at the intraspecies level.

## Background

**Figure 1:**
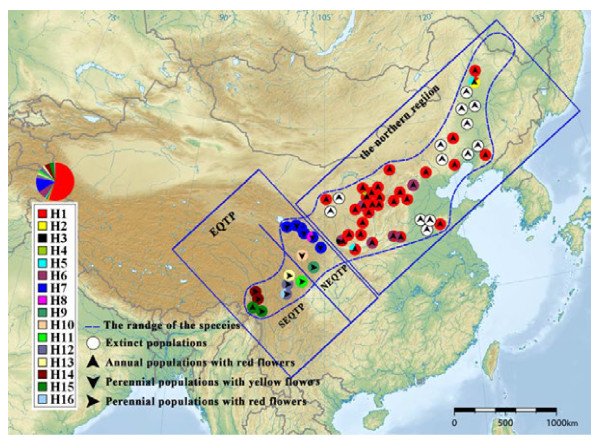
** The geographical distribution of*Incarvillea sinensis *and 16 haplotypes (H1-H16) found among 705 individuals.** QTP, NEQTP and SEQTP are the abbreviations of the Qinghai-Tibet Plateau, the northeastern Qinghai-Tibet Plateau and the southeastern Qinghai-Tibet Plateau respectively, as described in the materials and methods.

Patterns of genetic and geographical structure in natural populations have been strongly influenced not only by intrinsic factors, such as life histories and ecological traits, but also by extrinsic factors including habitats and historical events [1-4]. Population geneticists have been interested in illustrating the factors determining genetic structure over the long term [5,6]. During the past two decades, the phylogeographic histories of a broad spectrum of animal and plant species have been reconstructed, and phylogeographical analysis has proven to be a powerful method in searching for congruent geographical patterns of genetic variation and determining the roles that historical events have played in shaping the present spatial genetic structure of a species [3,7-20]. The elevation of the Qinghai-Tibetan Plateau and Quaternary glaciation were key geological events that significantly affected Asian topography, atmospheric circulation, and even global climate. These events influenced floristic distributions, and therefore it is important for biologists to seek out responses of organisms to the uplift of the Qinghai-Tibetan Plateau and the climatic fluctuations during glacial periods to better understand current species distributions [3,19,21-25]. Until very recently, numerous phylogeographical studies have outlined Quaternary evolutionary histories of European and Northern American floras (e.g., [3,16,26-29]), but relatively few investigations have focused on population divergence and phylogeography of plants in the Qinghai-Tibet Plateau [22-25,30-33]. In this study, we concentrated on elucidating the spatial genetic structure of an attractive herb, *Incarvillea sinensis* Lam., which is widely distributed from the southwest China through to the northeast China and the Russian Far East (Figure 1), whose biogeographic history was proposed to be closely related to the uplift of the Qinghai-Tibet Plateau and to Quaternary climate oscillations [34].

*Incarvillea sinensis* is the type species of *Incarvillea* Juss. (Bignoniaceae) including 16 species, and it also is the type of the subgenus *Incarvillea* including two species. Compared with other members of the genus centered in the Himalaya-Hengduan Mountains, this species has a broad range and altitudinal distribution (Figure 1). It is very rich in morphological variation, such as flower color, habit, and leaf characteristics, and its broad spectrum of morphological variants bewildered taxonomists in their attempts to propose intraspecific subdivisions; therefore, two classifications were established due to the discretionary priorities of morphological characteristics [35,36]. Based on habit (annual or perennial), Grierson [35] subdivided *I*. *sinsensis* into two subspecies: *I*. *sinensis* subsp. *sinensis* and subsp. *variabilis*. The former is an annual herb with simple roots, while the latter is a perennial herb with woody, branched roots and often bears the remains of the previous year’s stems. In another system described in Flora Reipublicae Popularis Sinicae [36], two variants were recognized, *I*. *sinensis* var. *sinensis* and var. *przewalskii*. The former is characterized by red flowers and the latter by yellow flowers. Both intraspecific classifications seemed reasonable given that straightforward characters (flower color or habit) were used to distinguish intraspecific taxa, but the annual populations with red flowers were confusing owing to the disjunct distribution between the northern region and the southernmost Lancangjiang Valley (Figure 1). The distribution was split due to the occurrence of perennial groups with yellow and red flowers in the middle of the entire species distribution.

After investigating all of the specimens of *Incarvillea sinensis* in CDBI, E, K, KUN, PE, and SZ, and exploring its distribution in China, we found that its variation in morphology was regional and that all populations can be classified into three types (Figure 1, Table 1). Type 1 is an annual with red flowers, distributed from the eastern Gansu Province to the Northeast China and the Russian Far East, with one population in the Lancangjiang Valley. Type 2 is a perennial with yellow flowers and is found in the western Gansu Province and the eastern Qinghai Province. Type 3 is a perennial with red flowers found in the southeastern Qinghai-Tibet Plateau, mainly in Sichuan Province. The morphological variations within *I*. *sinensis* can be correlated with their geographic distribution, while the eastern Qinghai-Tibet Plateau (QTP) contain all three morphological types, and appears to be the center of diversity for this species. We suspected that the spatial pattern of morphological characteristics likely dropped a hint to the potential divergence in genetics.

**Table 1:** **Details of samples and cpDNA haplotype distributions for 47 collections of*Incarvillea sinensis***

**PO**	**Localities**	**C**	**H**	**T**	**Lat. (N)**	**Long. (E)**	**Haplotypes(F)**
BT	Batang, Sichuan	R	P	15	30°03'54.2″	099°08'56.8″	H14(15)
CH	Chifeng, Neimenggu	R	A	15	42°45'38.2″	119°43'17.5″	H1(15)
CP	Changping, Beijing	R	A	15	40°18'17.1″	116°22'51.6″	H1(15)
DB	Bashong, Derong, Sichuan	R	P	15	29°19'41.6″	099°11'56.4″	H14(15)
DQ	Yanjing, Tibet	R	A	15	28°36'11.7″	098°45'32.7″	H15(15)
DR	Derong, Sichuan	R	P	15	28°47'39.1″	099°17'36.4″	H15(15)
DS	Danba, Sichuan	R	P	15	30°51'28.0″	101°51'15.2″	H16(6)/H12(9)
GA	Dingxi, Gansu	Y	P	15	35°38'11.0″	104°34'42.7″	H8(6)/H7(9)
GD	Diebu, Gansu	Y	P	15	33°56'44.0″	103°39'17.4″	H10(15)
GG	Lanzhou, Gansu	Y	P	15	35°56'54.1″	103°52'22.4″	H7(15)
GL	Tianshui, Gansu	Y	P	15	34°32'56.7″	105°43'19.4″	H7(15)
GW	Wenxian, Gansu	R	P	15	32°57'52.6″	104°39'07.2″	H9(15)
GY	Yongdeng, Gansu	Y	P	15	36°32'48.2″	102°56'13.4″	H7(15)
HL	Huanling, Shensi	R	A	15	35°33'19.1″	109°15'52.3″	H1(15)
HQ	Hequ, Shansi	R	A	15	39°23'06.8″	111°08'20.1″	H1(15)
HS	Hengshan, Shensi	R	A	15	37°58'39.1″	109°45'05.1″	H6(7)/H1(8)
HR	Heshui, Gansu	R	A	15	35°38'39.5″	107°53'19.4″	H1(15)
JB	Jingbian, Shensi	R	A	15	37°35'44.6″	108°46'29.3″	H1(15)
JC	Jinchuan, Gansu	R	A	15	35°20'59.7″	107°26'24.7″	H3(3)/H6(3)/H1(9)
JM	Jinchuan, Sichuan	R	P	15	31°14'04.1″	102°00'31.6″	H12(15)
JX	Jiaxian, Shensi	R	A	15	38°02'00.6″	110°28'57.4″	H1(15)
LS	Jincheng, Shansi	R	A	15	35°27'34.5″	113°03'55.3″	H6(7)/H1(8)
LT	Liancheng, Gansu	R	P	15	36°41'15.8″	102°44'44.4″	H7(15)
LX	Linxian, Shansi	R	A	15	38°07'56.8″	111°03'13.1″	H1(15)
LY	Laiyuan, Hebei	R	A	15	39°22'04.6″	114°42'31.0″	H6(15)
MD	Molidawaqi, Neimenggu	R	A	15	48°20'49.6″	124°26'51.4″	H1(15)
MG	Maixian, Sichuan	R	P	15	31°48'26.8″	103°50'29.0″	H11(15)
MS	Maerkang, Sichuan	R	P	15	31°54'57.6″	102°06'01.9″	H13(15)
MZ	Mizhi, Shensi	R	A	15	37°42'46.7″	110°10'45.4″	H1(15)
NZ	Zhuozi, Neimenggu	R	A	15	40°53'46.0″	112°35'02.8″	H1(15)
PS	Pingshan, Hebei	R	A	15	38°18'46.9″	113°59'41.4″	H1(15)
QQ	Qiqihaer, Heilongjiang	R	A	15	47°20'23.0″	123°57'44.7″	H5(5)/H2(8)/H1(2)
SD	Dabaodang, Shenmu, Shensi	R	A	15	38°40'30.8″	110°02'56.5″	H4(2)H1(13)
SS	Shenmu, Shensi	R	A	15	38°55'02.0″	110°28'26.8″	H1(15)
SZ	Suizhong, Liaoning	R	A	15	40°10'59.5″	119°48'49.7″	H1(15)
WG	Wutai, Shansi	R	A	15	38°40'04.7″	113°12'55.6″	H1(15)
XM	Xianyang, Shensi	R	A	15	34°20'09.6″	108°34'11.8″	H5(4)/H1(11)
XN	Xining, Qinghai	Y	P	15	36°37'48.4″	101°43'55.6″	H7(15)
XT	Xintai, Shandong	R	A	15	35°44'53.4″	117°28'45.6″	H1(15)
XX	Xingxian, Shansi	R	A	15	38°28'14.5″	111°14'07.5″	H1(15)
XY	Xiuyan, Liaoning	R	A	15	40°49'28.2″	123°07'18.5″	H1(15)
XZ	Xinzhou, Shansi	R	A	15	38°17'19.6″	112°42'31.8″	H1(15)
YA	Yanan, Shensi	R	A	15	36°29'47.4″	109°28'42.0″	H1(15)
YC	Yijinhuoluoqi, Neimenggu	R	A	15	39°21'45.4″	109°49'40.1″	H1(15)
YH	Yinchuan, Ninxia	R	A	15	38°42'27.1″	105°57'29.9″	H1(15)
YJ	Yongji, Shansi	R	A	15	34°50'40.7″	110°20'24.5″	H6(10)/H1(5)
ZZ	Zezhou, Shansi	R	A	15	35°30'29.7″	113°03'03.3″	H6(9)/H1(6)

PO, populations; C, flower colors; R, red flowers; Y, yellow flowers; H, habits; A, annual; P, perennial; T, number of individuals; Lat., latitude; Long., longitude; F, frequency of haplotype.

A study on the biogeography of *Incarvillea* suggests that its evolution may be related to the uplift of the Qinghai-Tibet Plateau. In addition, the continuous distribution of the subgenus *Incarvillea*, from the eastern Qinghai-Tibet Plateau to the Russian Far East, could partially be the result of the Quaternary expansion since part of its range was glaciated [34]. *Incarvillea sinensis* occupies almost the full range of the subgenus except in Mongolia. Considering the morphological variations correlative to geography, we proposed that the uplift of the Qinghai-Tibetan Plateau potentially resulted in the genetic variations between populations within and outside of the Qinghai-Tibetan Plateau. Furthermore, the Pleistocene glaciation possibly induced the extinction of some northern populations and the modern distribution likely resulted from the postglacial and interglacial expansion. In order to test the hypothesis, we examined the genetic diversity of populations within and outside the Qinghai-Tibet Plateau and identified potential genetic lineages within this species.

Since the colonization of new habitats occurs through seeds, and chloroplast DNA is generally maternally inherited in angiosperms (meaning that the plastid genome is moved only by seeds) [13], chloroplast DNA (cpDNA) markers provide information on past changes in species ranges that are unaffected by subsequent pollen movements [37]. We investigated patterns of cpDNA diversity in *Incarvillea sinensis* using phylogeographical methods that can indicate how historical events such as range fragmentation, range expansion, and long distance dispersal, as well as current levels of gene flow, have influenced present-day distributions. The goals of this study are to provide new insights into the population genetic structure and responses of *I*. *sinensis* to geological events and reveal the imprints of geological events on the spatial pattern of the genetic diversity.

## Results

### Sequence variation and haplotype distribution

A matrix of 1,396 characters was obtained from concatenated alignments of *trn*H- *psb*A (377 sites) and *trn*S- *trnf*M (1,019 sites) sequences from 705 individuals representing 47 collections, in which 38 polymorphic sites were confirmed by sequencing in both directions. Sequences with polymorphisms between each other were deposited in GenBank (Accession No. JQ858492-JQ858511). Of these polymorphisms, five were indels (insertions and deletions). Two indels were single nucleotides and the other three were six bp, five bp and seven bp long respectively. In subsequent analysis, indels with length more than one base were considered as a single mutation. All five indels were coded as binary characters, 1 s and 0 s, so that the matrix was shortened to 1381 characters in length: 372 sites from *trn*H- *psb*A and 1,009 sites from *trn*S- *trnf*M. The adjusted matrix included 23 variable sites, five of which were due to the presence of indels, and all of variable sites were informative and involved in all analysis. All of the sequences from the 47 localities contained 16 unique haplotypes when indels were included (Table 2). Haplotypes and their frequency from each locality are shown in Table 1 and their spatial pattern is portrayed in Figure 1.

**Table 2:** **Polymorphic sites of 16 haplotypes found for*Incarvillea sinensis*based on two cpDNA segments**

**NP**	***trn*H- *psb*A(1–372)**										***trn*S- *trnf*M(373–1381)**												
	**8**	**9**	**1**	**1**	**1**	**1**	**2**	**2**	**2**	**2**	**4**	**9**	**9**	**9**	**9**	**1**	**1**	**1**	**1**	**1**	**1**	**1**	**1**
	**0**	**7**	**6**	**6**	**9**	**9**	**5**	**6**	**8**	**9**	**4**	**2**	**4**	**4**	**4**	**0**	**0**	**0**	**1**	**1**	**2**	**3**	**3**
			**1**	**5**	**5**	**6**	**4**	**9**	**9**	**8**	**8**	**0**	**4**	**5**	**6**	**0**	**3**	**6**	**2**	**5**	**8**	**7**	**7**
																**4**	**5**	**2**	**4**	**0**	**5**	**2**	**5**
H1	A	C	T	G	-	-	-	G	A	G	G	G	C	A	A	T	A	G	G	*	-	T	G
H2	A	C	T	G	-	-	-	G	A	A	G	G	C	A	A	T	A	G	G	*	-	T	G
H3	A	C	T	G	-	-	-	G	A	G	G	G	A	A	A	T	A	G	G	*	-	T	G
H4	A	C	T	G	-	-	-	G	A	G	G	A	C	A	A	T	A	G	G	*	-	T	G
H5	A	C	T	G	-	-	-	G	A	G	G	G	C	C	A	T	A	G	G	*	-	T	G
H6	A	C	T	G	-	-	-	G	A	G	G	G	C	C	C	T	A	G	G	*	-	T	G
H7	G	T	T	T	A	-	-	T	G	G	G	G	C	A	A	C	A	G	T	-	†	C	C
H8	G	T	T	T	A	-	#	T	G	G	G	G	C	A	A	C	A	G	T	-	†	C	C
H9	G	T	T	T	A	-	-	A	G	G	G	G	C	A	A	C	A	G	T	-	†	C	C
H10	G	T	T	T	A	A	-	A	G	G	G	G	A	A	A	C	A	G	T	-	†	C	C
H11	G	T	T	T	A	-	-	T	G	G	G	G	C	C	A	C	A	G	T	-	†	C	C
H12	G	T	T	T	A	-	-	T	G	G	G	G	C	C	A	C	A	C	T	-	†	C	C
H13	G	T	C	T	A	-	-	T	G	G	G	G	C	C	A	C	A	C	T	-	†	C	C
H14	G	T	T	T	A	-	-	T	G	G	G	G	C	C	C	C	A	C	T	-	†	C	C
H15	G	T	T	T	A	-	-	T	G	G	G	G	C	C	C	C	C	C	T	-	†	C	C
H16	G	T	T	T	A	-	-	T	G	G	A	G	C	C	A	C	A	C	T	-	†	C	C

NP, nucleotide position; # = CTTCTA * = TATTA † = GATTAGG.

### Genealogical relationships among haplotypes of *Incarvillea sinensis*

**Figure 2:**
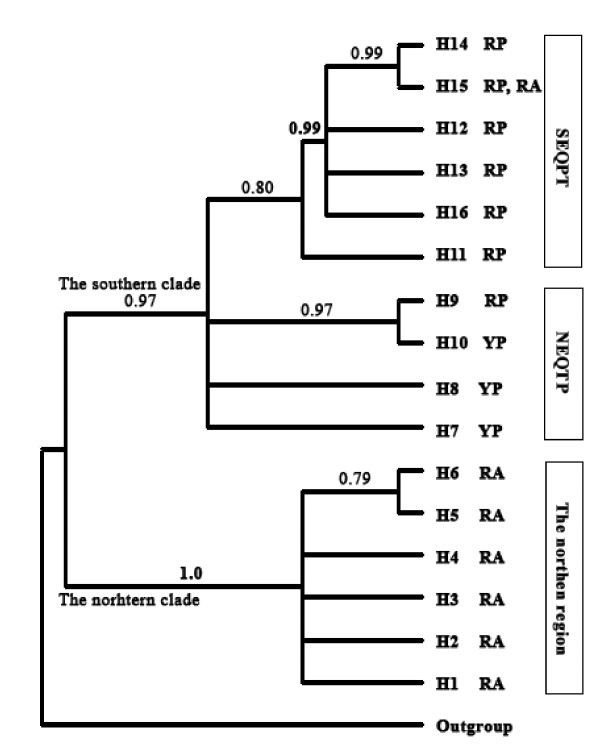
** Phylogram among cpDNA (*trn*S-*trnf*M and*trn*H-*psb*A regions) haplotypes of*Incarvillea sinenesis*resulting from Bayesian analysis.** Numbers above the branches are posterior probabilities of nodes. Posterior probabilities of Bayesian run without assuming the molecular clock was enforced. RA: red flowers, annual; RP: red flowers, perennial; YP: yellow flowers, perennial; SEQTP: the southeastern Qinghai-Tibet Plateau; NEQTP: the northeastern Qinghai-Tibet Plateau.

In our phylogenetic analysis, a data set including haplotype and outgroup sequences was used to reconstruct the relationships among the haplotypes. This matrix was 1,447 sites in length, and contains 38 informative sites. The consensus tree resulting from Bayesian analysis is presented in Figure 2. The ingroup sequences clustered into two major clades, the southern and northern clades, concordant with geography (Figures 1, 2). The northern clade accommodated six haplotypes (H1-H6) from the northern distribution, and 10 haplotypes (H7-H16) from the southern distribution were nested in the southern clade. Moreover, a subclade was formed within the southern clade to accommodate six haplotypes (H11-H16) from the southeastern Qinghai-Tibet Plateau, situated in the southernmost range of *I*. *sinensis*.

**Figure 3:**
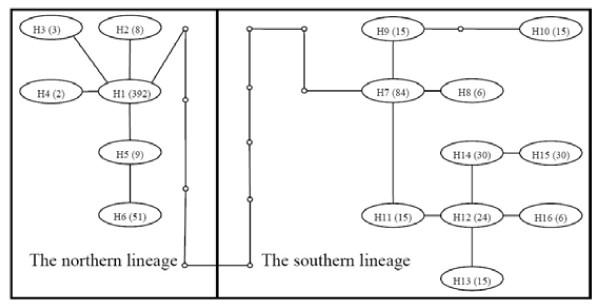
** The parsimonious network of cpDNA Haplotypes of*Incarvillea sinensis*inferred using the TCS program.** Every haplotype is identified by a number and each branch represents a mutational step with intermediate missing haplotypes represented by small and empty dots. The numbers in the parentheses are the frequencies of the haplotypes.

Compared with the phylogenetic tree, the haplotype network with 95% connection constructed using the TCS program was better resolved in genealogical relationships among the haplotypes, but the network showed a loop indication ambiguous connections involving haplotypes H1, H5, H7, and H11. Following the rules of Crandall & Templeton [38] in combination with the genetic distances between haplotypes and the phylogenetic tree, the loop was resolved and the network was shown in Figure 3. Two lineages from the southern and northern regions were connected by 11 mutations. The northern lineage contained six haplotypes (H1-H6) from the northern region (that is, the northern clade in the phylogenetic tree), and the southern lineage contained 10 haplotypes (H7-H16) from the eastern Qinghai-Tibet Plateau (that is, the southern clade in the phylogenetic tree).

The net averaged population genetic distance per site between two regional lineages calculated using Arlequin was 0.00879, and the divergence of these two major clades within this species was dated at 4.396 MYA.

### Population genetic parameters and phylogeographical structure

The total gene diversity *h*_T_ was 0.677 and the average within-population gene diversity *h*_S_ was 0.106 at the species level. The indices of population structure *G*_ST_ and *N*_ST_ were 0.843 and 0.975, respectively. The permutation test showed that *N*_ST_ was significantly larger than *G*_ST_ (*P* < 0.05). The *G*_ST_ and *N*_ST_ at the regional level implicated that significant structures existed in the SEQPT, NEQTP and their combined regions, but not in the northern region. Genetic parameters at different levels are listed in Table 3.

**Table 3:** **Statistic summary of genetic diversity and population subdivision of*Incarvillea sisnensis***

**Groups**	**Number of populations**	**Number of haplotypes**	**Genetic diversity and divergence**				
			**h_s_**	**h_t_**	**G_st_**	**N_st_**	**P-value**
NEQTP	8	4	0.064	0.536	0.880	0.938	<0.05
SEQTP	7	5	0.064	0.907	0.929	0.960	<0.05
EQTP	16	10	0.064	0.870	0.926	0.973	<0.05
The northern	31	6	0.127	0.282	0.548	0.546	<0.05
Total	47	16	0.106	0.677	0.843	0.975	<0.05

NEQTP: the northeastern Qinghai-Tibet Plateau; SEQTP: the southeastern Qinghai-Tibet Plateau; QTP: the Qinghai-Tibet Plateau.

The analysis of molecular variance (AMOVA) of the data from the two cpDNA fragments indicates the presence of a strong differentiation in genetics among the lineages and regions (Table 4). When populations were grouped by three geographical regions, a statistically significant structure was detected by AMOVA. The comparison among the three regions shows that 93.94% (P < 0.001) of the genetic variations occurred between these regions. Only 4.7% (P < 0.001) of the variance was distributed within each of these regions, and less variance (1.36%, P < 0.001) can be attributed to the variations within the populations. When populations were grouped by two lineages, once again, the results show a similar pattern. Once the populations were grouped by subspecies and variants on taxonomy, the genetic variations between the groups decreased, and the remaining variations from the other two sources increased accordingly. The least amount of variance was contributed by the variations within populations, and this value is less than 1.5%.

**Table 4:** **Results from Analysis of molecular variance of data from two cpDNA fragments of*Incarvillea sinensis***

**Grouped by**	**Source of variations**		
	**Among groups**	**Among populations within groups**	**Within populations**
Two lineages	91.59^*^	7.16^*^	1.25^*^
Three regions	93.94^*^	4.70^*^	1.36^*^
Two subspecies	86.95^*^	11.77^*^	1.28^*^
Two variants	58.41^*^	40.18^*^	1.41^*^

^*^ P < 0.05.

### Correlation between genetic and geographic distances

**Figure 4:**
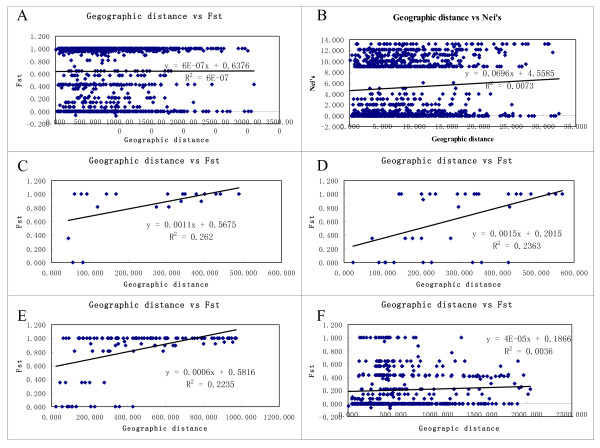
** Scatterplots representing relationships between genetic distance (*F***_**ST**_**) and geographic distance (Km) at species and regional levels.** A: species level, *r* = 0.001, *P* = 0.480; B: species level, r = 0.083, p = 0.034; C: the southeastern Qinghai-Tibet Plateau, *r* = 0.512, P = 0.006; D: the northeastern Qinghai-Tibet Plateau, *r* = 0.486, *P* = 0.025; E: the Qinghai-Tibet Plateau, *r* = 0.473, *P* < 0.001; F: the northern region, *r* = 0.06, *P* = 0.249; F: N region except the recently isolated and small populations, *r* = 0.29, *P* = 0.011.

The Mantel test quantified the correlations between the two distance matrices, allowing for the determination of the relationships between the genetic and the geographical distances. The results of this test did not show a significant effect of isolation by distance at the species level (*r* = 0.001, *P* = 0.48, Figure 4A). The distributions of haplotype showed that only six haplotypes were detected in 31 populations from the northern region, and 20 of them possessed the same haplotype (H1), so we performed this test once again after replacing the *F*_st_s with pairwise Nei’s genetic distances. The positive correlation between genetic difference and geographic distance (isolation by distance) was encountered in the species (*r* = 0.083, *P* = 0.034, Figure 4B). Furthermore we found significantly positive and monotonic relationships across all of the pairwise *F*_ST_ measures and spatial distances separating the populations of regions in SEQPT, NEQTP, and their combination (Figure 4C, D, and E), whose pattern of scatterplots were consistent with Case I proposed by Hutchison & Templeton [39]. This pattern describes the scenario of regional equilibrium between gene flow and drift (i.e., isolation by distance). In contrast, the null hypothesis of the regional equilibrium was rejected in populations from the northern region, and the scatterplot approximated case Ш of Hutchison & Templeton [39], indicating that drift is more influential than gene flow(*r* = 0.06, *P* = 0.249, Figure 4E).

## Discussion

### Conflict between the phylogeny and intraspecific classifications previously proposed

We found neither of the intraspecific subdivisions of the two subspecies and the two variants was supported by phylogenetic analysis, although the species was proven to be monophyletic by the cpDNA *trn*H- *psb*A sequence (not shown). Because the haplotype H15 was detected in the DQ population, the annual DQ population with red flowers phylogenetically clustered together with all of the other perennial populations with red flowers from the southeastern Qinghai-Tibet Plateau, not together with the annual populations with red flowers from the northern region (Table 1, Figure 1 &2). According to the system described by Wang et al. [36], the DQ population should be classified into *I*. *sinensis* var. *sinensis*, while the other populations from the southeastern Qinghai-Tibet Plateau should be classified into *I*. *sinensis* var. *przewalskii*. Likewise, the perennial GW population (haplotype H9) with red flowers was most closely related to the perennial populations with yellow flowers from the northeastern Qinghai-Tibet Plateau (Table 1, Figure 1 &2). Based on the Grierson’s system [35], the former should be classified into *I*. *sinensis* subsp. *sinensis*, while the latter should be *I*. *sinensis* subsp. *variabilis*.

Our study provides evidence in support of parallel evolution of morphological characteristics in allopatric populations (Figure 2). Character convergence within this species apparently resulted from adaptation to similar niches. From the genetic data, we found no evidence warranting the suggestion of monophyly for any of the recognized taxa. We therefore propose to view this species as a species complex that should not be subdivided into intraspecific units in taxonomy.

### Genetic diversity and structure at species and regional levels

The mean within-population genetic diversity is low compared with the total diversity at both species and regional levels. The AMOVA results also showed that most of the genetic diversity was attributed to the among-population effects. The global indices of population differentiation were very high, up to 0.843 and 0.975 for *G*_ST_ and *N*_ST_, respectively. When *N*_ST_ is significantly higher than *G*_ST_, this usually indicates the occurrence of a phylogeographical structure [40]. Through random permutations, a significant difference between these two measures was detected within this species. On the other hand, 16 haplotypes were clustered into two lineages or three groups harmonious with geography (Figure 2 &3), and none of them was shared among the regions (Figure 1). These results reveal that the cpDNA variation of this species is highly structured.

The southern clade consists of four haplotypes from the NEQTP and six haplotypes from the SEQTP. Two predominant haplotypes (H7 & H8) are widely distributed in NEQTP, except in Wenxian and Diebu in Gansu Province, and the remaining eight haplotypes are relatively rare and endemic to one valley or locality (Figure 1, Table 1). The indices of genetic differentiation show that geographic structure exists both in the lineage and in the subdivided regions (SEQTP and NEQTP regions, Table 3). The northern clade is distributed in the northern region where six haplotypes (H1-H6) are scattered erratically, and no geographic structure was detected (*N*_ST_ < *G*_ST_). We note that both the differentiation index (*G*_ST_) and the diversity index (*h*_T_) of the sorthern clade is higher than those of the southern clade. The degree of subdivision of the cpDNA diversity (*G*_ST_) reflects the dispersal ability of the species and the effect of long-term range fragmentation, while the low *G*_ST_ value is indicative of high levels of gene flow through seeds [13]. Once capsules of *Incarvillea sinensis* split, the seeds are ejected and the seed wings contribute to seed dispersal by wind. In the southern region accommodating the southern clade, orogenesis resulted in range fragmentation, and populations of *I*. *sinensis* were distributed in valleys, except for those populations with haplotypes H7 and H8 (Figure 1). The connections between these populations were severed by towering ridges and peaks. As a result, efficient seed dispersal was reduced and the gene flow through seed dispersal was confined within the valleys, or between the neighboring populations.

In contrast, the analogous limiting factors of seed movement did not exist in the northern region, and we could not identify the existence of a significant geographic structure in the northern clade (*G*_ST_ > *N*_ST_). Distantly related haplotypes were more often found in the same populations, as case 3 proposed by Pons & Petit [40], which indicates that the relative geographic distribution of the haplotypes may have nothing to do with their genetic distances, in this case old lineages have had ample time to become geographically redistributed since they first appeared as products of mutation. Based on the comparison of the indices of genetic diversity and differentiation between two lineages, we consider the topographical barriers in gene flow through seed dispersal to be the most likely factors leading to the higher divergence in the southern clade. The diverse niches in the eastern Qinghai-Tibet Plateau created a wide spectrum of habitats to accumulate and accommodate new mutations.

### Distinctive evolutionary histories between lineages

The two major cpDNA lineages were revealed by means of reconstructing the phylogenetic tree and the haplotype network, which can be recognized by a connection of 11 mutations. The deep gap between these two clades indicates the early origin of the corresponding lineages in the evolution of the species, and the long-standing separation thereafter. We dated the divergence between the two lineages at approximately 4.4 MYA, which corresponds to the early Pliocene. The uplift of the Himalaya-Hengduan massifs was a complicated geological event, and the chronology of this process is still under debate [41-45]. It is believed that the plateau underwent its most recent dramatic uplift after the early Pliocene [43,44]. The two lineages of *Incarvillea sinensis* most likely originated because of decreased effective gene flow due to topographic isolation after orogenesis and long term adaptation to the distinct habitats (if their non-overlapping distributions and unshared haplotypes are taken into consideration). The long evolutionary history allowed this species to accumulate not only mutations relative to diversity, but also high differentiation between regions.

For the northern lineage, the long history should be enough for localized gene flow to interact with drift to produce a pattern of isolation by distance across the region (i.e., regional equilibrium), but the correlation estimation of pairwise genetic and geographical distances show that drift was much more influential than gene flow on the distribution of genetic variability (Figure 4E). Populations were not at equilibrium, either because conditions required or the populations themselves have not existed long enough for regional pattern of isolation-by-distance to have been achieved [39,46]. The northern lineage was composed of six haplotypes detected in 31 collections from the northern region. The basal and predominant haplotypes (H1) was distributed randomly and the other five haplotypes were concentrated on the haplotype H1 in the network (Figure 3). The starlike shape of the network of the six haplotypes reflected the extremely low level of the sequence divergence and the low frequency of rare haplotypes. The lack of regional equilibrium, the lower diversity and starlike shape network of the northern lineage likely indicated that possible extinction during the glacial and the interglacial and postglacial recolonization.

On the contrary, within the southern clade, including all haplotypes from the eastern Qinghai-Tibet Plateau, a significant association between the genetic variability and the geographical distribution was revealed by the Mantel test. The Mantel tests also showed that there was a significant effect of isolation by distance, which means that a regional equilibrium was reached and that more closely situated populations tended to communicate and be more genetically similar to one another [47]. Obviously, gene flow was more easily accomplished on the alpine platform than in fragmented landscapes, such as the southeastern Qinghai-Tibet Plateau. We conclude that the populations from the southeastern Qinghai-Tibet Plateau suffered severe isolation owing to orogenesis, which significantly reduced seed dispersal, so that gene flow through the seeds was confined between neighboring populations within areas without strict topographical barriers. Allopatric fragmentation became a theme throughout the evolutionary history of the southern lineage. Significant ridges and peaks of the local landscape formed permanent spatial barrier that intensified and fixed genetic isolations.

## Conclusions

We detected two lineages connected by a deep gap within the species, and significant difference in genetic diversity between them. The significant genetic divergence concordant with geography and the features of genetic diversity seemed to reveal the imprints of geological events on the plant evolution. We preferred the significance of the uplift of the Qinghai-Tibet Plateau and the Quaternary Glacial in the distribution of the genetic diversity and differentiation of *Incarvillea sinensis*, as mentioned by Chen et al. [34].

The Qinghai-Tibet Plateau and its adjacent areas have been listed as one of the world’s biodiversity hotspots [48,49]. Two processes may contribute to the formation of biodiversity centers: the elevation of local speciation rates (the center of origin hypothesis) and a greater accumulation of species formed elsewhere (the center of accumulation hypothesis). Available studies in this area have focused mainly on traditional taxonomy, botanical inventory, and phylogeny-based species radiation [50-53], and as evidence mounts, it becomes increasingly clear that both the origin and the accumulation hypotheses have acted in concert to form this biodiversity hotspot [51,54-59]. Only a few plant species in this region, however, have been phylogeographically studied (e.g. [22-25,30,32,33]). In the absence of phylogeographical investigations on a multitude of plants, it is very difficult to uncover the common microevolutionary patterns of the extant organisms within this area. The pattern of genetic variation within the widely distributed *Incarvillea sinensis* populations, however, offers clues to the mechanisms of the evolutionary of the diversity of plants in the Qinghai-Tibet Plateau. Our present study not only shows that the spatial genetic structure within this species likely is the result of tectonic events and long term adaptation to distinct habits, but also supports an evidence on the higher diversity in the Qinghai-Tibet Plateau influenced by origins.

## Methods

### Sample collections

We conducted extensive fieldwork following herbarium specimen records of *Incarvillea sinensis* from CDBI, E, K, KUN, PE, and SZ. A number of populations, in some locations, became extinct due to habitat destruction. Numerous specimens were collected in Henan, Shandong, Inner Mongolia, Jilin, Liaoning, and Heilongjiang provinces during the 1920s–1950s. Subsequently, fewer collections have been recorded; and no new specimens were recorded since 1985. We were able to obtain only eight samples in these areas, seven of which (i.e., CH, MD, QQ, SZ, XT, XY, and YH, see Table 1) were isolated and small in size. In this study, we sampled 705 individuals from 47 populations distributed in China (Figure 1, Table 1) covering the entire range of the species, except for the Russian Far East, where only a few collections were recorded. Leaves were dried using silica-gel. A modified CTAB extraction protocol was used to extract DNA from the dried leaf tissue [60].

### Chloroplast DNA amplification and sequencing

Two regions of cpDNA, *trn*H- *psb*A and *trn*S- *trnf*M were amplified and sequenced. The mixture for polymerase chain reactions consisted of a total volume of 25 μL containing 2.5 μL of MgCl_2_-free buffer, 1.5 μL of MgCl_2_ solution (25 mM), 0.5 μL of dNTPs (2.5 mM each), 1.25 μL of each primer (10 μM), 1 U *Taq* polymerase, 1 μL of template ( *c*. 50–100 ng double-stranded DNA), and 17.25 μL of sterile water.

The *trn*H- *psb*A segment was amplified using the primers *trn*H [61] and *psb*A [62], and *trn*H was used for sequencing. The PCRs consisted of an initial denaturation at 94°C for 3 min, followed by 30 cycles of 30 s at 94°C (denaturation), 30 s at 52°C (annealing), and 30 s at 72°C (extension), and concluding with a final extension period of 7 min at 72°C.

The *trn*S- *trnf*M fragment was amplified using the *trn*S and *trnf*M primers [63] and both primers were used in sequencing. The amplification parameters for this segment were 94°C, 3 min; 30 cycles (94°C, 30 s; 52°C, 30 s; 72°C, 1 min); 72°C, 7 min.

The PCR products were purified with DNA-purification kits (Shenggong, Shanghai). The *trn*H- *psb*A and *trn*S- *trnf*M PCR products were sequenced in both directions using the ABI BigDye version 3.1 terminator cycle sequencing chemistry, with sequences read by an ABI 3730 Genetic Analyser (Applied Biosystems).

### Phylogenetic analysis

Sequences were aligned using ClustalX (version 1.81, [64]), and indels were coded as substitutions following Caicedo & Schaal [65] using a '1' for present and '0' for missing. Haplotypes were identified using DnaSP (version 3.0, [66]). We performed phylogenetic analysis on haplotypes of *Incarvillea sinensis* using MrBayes V3.1.2 [67]. Based on the phylogenetic relationships among species of the genus, the outgroup *Incarvillea arguta* was used to root the phylogenetic trees, which is similar to *I*. *sinensis* on morphology such as the flower, fruit and habit. We ran 500,000 Markov chain Monte Carlo (MCMC) generations for the Bayesian analysis, with a sampling frequency of 1 in 100 generations. The consensus tree was constructed from trees saved after reaching the stationarity (the density plot took a normal shape after ca. 6000 generations). Tree-building methods might not always be the most appropriate way to represent genealogical relationships among haplotypes within a species, owing to the possibly shallow genetic divergence [68]; thus, a haplotype network was built using the TCS program [69] with the statistical parsimony method described by Templeton et al. [70] and Crandall et al. [71]. Ambiguities in the haplotype network were resolved following the recommendations of Crandall & Templeton [38].

### Genetic diversity and divergence

The genetic structure was evaluated by the analysis of molecular variance [72] using ARLEQUIN 3.11 version [73], partitioning the genetic diversity within populations, among populations within groups, and among groups. Significance was assessed after 1,023 permutations. Populations were grouped by clades in phylogenetic trees, intraspecific taxonomic categories (subspecies and variants based on two taxonomic systems), and spatial regions. The geographic regions used for the AMOVA were: (1) the southeastern Qinghai-Tibet Plateau (SEQTP), including the southeast Tibet and the Sichuan Province; (2) the northeastern Qinghai-Tibet Plateau (NEQTP), including the Qinghai Province and the western Gansu Province; and (3) the northern area, including all of the remaining distributions outside the Qinghai-Tibet Plateau (Figure 1). The SEQTP and NEQTP regions composed the southern region.

We calculated within-population diversity (*h*_S_), total diversity (*h*_T_), and the level of population differentiation (*G*_ST_ and *N*_ST_) at the species and regional levels. The occurrence of a significant phylogeographical structure was inferred by testing whether *G*_ST_ (the index that only considers haplotype identities) and *N*_ST_ (the index that takes into account a measure of haplotype divergence) were significantly different using 1000 permutations in PERMUT [40,74].

Traditional *F*_ST_ and *G*_ST_ estimates can not distinguish different genetic structures with similar *F*_ST_ values because the *F*_ST_ is a compound product of gene flow and drift. In response, Hutchison & Templeton [39] proposed a method to evaluate the relative historical influences of gene flow and drift on regional population structure by constructing regional scatterplots of *F*_ST_ on geographical distances and calculating the correlation coefficients describing the relationship between them. Following this method, the *F*_ST_ values between pairwise populations were calculated using ARLEQUIN. Scatterplots of the *F*_ST_ on geographical distances were constructed, and correlation coefficients were calculated along with the significance of correlation by performing GenAlEx6 [75], in which the genetic distance matrices (pairwise *F*_ST_) were compared with the matrix of geographical distance by means of a simple Mantel test to detect isolation by distance and to evaluate the relative influences of gene flow and drift on the regional population structure. We used 999 random permutations to test for the Mantel statistic significance.

### Dating the divergence between the major groups

The net averaged population genetic distance, *D*_A_ *= d*_*i*XY_ - (*d*_*i*X_ + *d*_*i*Y_)/2 [76], accounts for the polymorphism within populations, and is thus proportional to the time since the divergence of two reciprocally monophyletic groups, assuming homogeneity of the mutation rates across the lineage [77]. To date approximately the divergence of high-level clusters reconstructed using phylogenetic and statistic parsimony analysis, we estimated the net pairwise divergence per site (*d*_A_) using ARLEQUIN, and then calculated the divergence time following the formula T = *d*_A_/2 μ [78]. In seed plants, the approximate evolutionary rate of noncoding spacer or introns of cpDNA was estimated at 1.01 × 10^-9^ substitutions per site per year [79], which can be used to estimate divergence time for taxa without fossil records (e.g., [24,80]; etc.). We also used this rate as the proximate evolutionary rate of the *trn*H- *psb*A and *trn*s- *trnf*M fragments owing to the lack of any fossils for this group to use as calibration points.

## Authors’ contributions

Shaotian Chen carried out field work, the molecular genetic studies, the statistical analysis, and drafted the manuscript. Yaowu Xing and Tao Su participated in sample collections and sequencing. Zhekun Zhou designed the study. David L. Dilcher and Douglas E. Soltis polished the manuscript, and David L. Dilcher helped to conceive of the manuscript. All authors read and approved the final manuscript.
